# Post-gastrectomy anemia and ferritin dynamics: key determinants of prognosis and clinical management in patients with gastric cancer

**DOI:** 10.3389/fonc.2025.1487477

**Published:** 2025-03-14

**Authors:** Eun Young Kim, Kyo Young Song, Dong Jin Kim

**Affiliations:** ^1^ Department of Surgery, Uijeongbu St. Mary’s Hospital, College of Medicine, The Catholic University of Korea, Seoul, Republic of Korea; ^2^ Department of Surgery, Seoul St. Mary’s Hospital, College of Medicine, The Catholic University of Korea, Seoul, Republic of Korea; ^3^ Department of Surgery, Eunpyeong St. Mary’s Hospital, College of Medicine, The Catholic University of Korea, Seoul, Republic of Korea

**Keywords:** anemia, ferritin, hemoglobin, gastrectomy, gastric cancer

## Abstract

**Objective:**

This study identified the trends and clinical significance of anemia and ferritin status 1 year postoperatively in patients with long-term survival and analyzed clinicopathological factors and preoperative nutritional/inflammatory conditions associated with anemia of chronic disease (ACD) development.

**Methods:**

Between March 2009 and December 2018, 2,976 patients who underwent curative gastrectomy for gastric cancer without recurrence or mortality within postoperative 1 year were included. The patients were categorized into four groups; non-iron deficiency without anemia, iron deficiency without anemia, iron deficiency anemia (IDA), and ACD based on postoperative 1 year ferritin and hemoglobin.

**Results:**

The overall incidence of anemia was 36.5% (n=1,086). The prevalence of IDA and ACD was 12.7% (n=377) and 23.8 (n=709), respectively, at postoperative 1 year. Patients with ACD were significantly older, had higher ECOG, increased early complications, and were at a more advanced stage than the other groups. The overall survival (OS) of ACD was significantly lower than that of the other groups (*p* < 0.001), especially for stages I and III. The presence of ACD was a significant risk factor for overall (hazard ratio [HR] = 1.832, *p* < 0.001), disease-free (HR= 1.714, *p* = 0.003), and cancer-specific (HR= 1.690, *P* = 0.015) survival. Older age, advanced disease stage, low preoperative prognostic nutritional index, preoperative anemia, and early postoperative complications were significant risk factors for ACD.

**Conclusions:**

Relationship between ferritin and Hb at postoperative 1 year is a significant prognostic factor for survival in patients with gastric cancer. Particularly, ACD may be a specific predictor of gastric cancer. Therefore, clinicians need to pay attention to ACD status and prevent the risk factors for its development during long-term postoperative follow-up.

## 1 Introduction

According to the 2021 Cancer Statistics of Korea, gastric cancer is one of the fourth common cancers and the fourth leading cause of cancer-related deaths in Korea (1) whereas it is the fifth most common cancer and the fifth leading cause of cancer-related deaths worldwide based on GLOBOCAN 2022 (2).

Traditionally, curative gastrectomy has remained the cornerstone of treatment for resectable gastric cancer (3). However, anemia has emerged as a prevalent post-gastrectomy complication, often attributed to iron, vitamin B12, or folate deficiencies, either in isolation or synergistically (4, 5). These deficiencies typically arise from factors such as malabsorption, diminished dietary intake, or chronic gastric mucosal bleeding (4, 5).

Several studies have indicated a potential relationship between post-gastrectomy anemia, nutritional deficiencies, and unfavorable prognostic outcomes (6, 7). However, most prior investigations have predominantly relied on hemoglobin (Hb) levels as the sole indicator to assess the prevalence of anemia following gastrectomy (7, 8). Furthermore, studies simultaneously examining the occurrence of iron-deficiency anemia (IDA) and anemia of chronic disease (ACD) in post-gastrectomy state are scarce, and the correlation between these types of anemia and gastric cancer prognosis has been scarcely investigated.

Ferritin, a pivotal iron-binding protein, reflects the iron reserves within the body (9). Recent findings have underscored its pivotal involvement in critical processes including cancer cell proliferation, angiogenesis, immunosuppression, carcinogenesis, and treatment resistance (10). Elevated serum ferritin levels have consistently been correlated with poorer survival outcomes across various malignancies, such as lung, colorectal, breast, pancreatic, and hepatocellular carcinomas (11–14). However, the prognostic significance of serum ferritin levels in gastric cancer remains controversial. Literature regarding the prognostic significance of serum ferritin levels, specifically in patients with gastric cancer undergoing gastrectomy, is scarce (15, 16).

Therefore, this study aimed to classify post-gastrectomy patients (long-term survivors of gastric cancer) based on the occurrence patterns of anemia and ferritin levels at postoperative 1 year, and to assess their clinical impact on gastric cancer prognosis. Additionally, the clinicopathological factors, preoperative nutritional status, and inflammatory conditions associated with ACD were analyzed.

## 2 Materials and methods

Medical records of patients with gastric cancer who underwent curative gastrectomy at Seoul St. Mary’s Hospital and Yeouido St. Mary’s Hospital between March 2009 and December 2018 were reviewed. Patients with remnant gastric cancer who underwent total gastrectomy, hematologic disease, cancer recurrence, or death within postoperative 1 year; underwent Whipple procedure; and those who had no postoperative 1 year laboratory data were excluded. After exclusion, 2,976 patients were enrolled in this study. This study was approved by the Institutional Review Board (XC22RIDI0045) and was performed in line with the principles of the Declaration of Helsinki.

### 2.1 Classification and measurement of biochemical and anthropometric variables

Anemia was defined as Hb < 12 g/dL in women and < 13 g/dL in men according to the World Health Organization (WHO) criteria. Iron deficiency was defined as a serum ferritin level < 15 ng/mL, regardless of anemia. ACD was defined as anemia with serum ferritin level ≥ 15 ng/mL. The patients were categorized into four groups; non-iron deficiency without anemia, iron deficiency without anemia (ID), IDA, and ACD based on postoperative 1 year ferritin and Hb level. The clinicopathological features and long-term prognoses of these groups were comparatively analyzed.

The platelet-to-lymphocyte ratio (PLR) was defined as platelet count divided by lymphocyte count. The neutrophil-to-lymphocyte ratio (NLR) was defined as neutrophil count divided by lymphocyte count. The prognostic nutritional index (PNI) was calculated using the following formula: 10 × serum albumin level (g/dL) + 0.005 × peripheral blood lymphocyte count. Patients were classified into high and low groups according to the median PNI value.

Body mass index (BMI) was calculated using the following formula: weight (kg)/height^2^ (m^2^). Patients were classified according to BMI, based on the WHO definition of obesity for Asians, as follows: underweight (BMI < 18.5 kg/m^2^), normal/overweight (18.5 ≤ BMI < 25.0 kg/m^2^), or obese (BMI ≥ 25.0 kg/m^2^).

### 2.2 Statistical analysis

Continuous and categorical variables were compared using the independent t-test and chi-square test, respectively. Kaplan–Meier curves were used to estimate overall survival (OS), disease-free survival (DFS), and cancer-specific survival (CSS). The survival rates of the groups were compared using log-rank test. A Cox regression model was used to identify variables influencing OS, DFS, and CSS. Logistic regression analysis was performed to assess the factors independently associated with the presence of ACD. In all analyses, *P* < 0.05 was considered to indicate statistical significance. All statistical analyses were performed using the SPSS software (version 18.0; SPSS Inc., Chicago, IL, USA).

## 3 Results

### 3.1 Clinicopathological characteristics and prevalence of anemia

The clinicopathological characteristics and prevalence of anemia are summarized in **Table 1**. Of the 2,976 patients, 1,912 (642.%) were male. Majority were presented with ECOG 0 or 1 (n = 2870; 96.4%). Overall, 2,358 (79.2%) and 2,474 (83.1%) patients underwent partial gastrectomy and duodenal non-passing anastomosis, respectively. A number of postoperative complications within 30-day after operation was 644 (21.6%). The prevalence of stage I, II, and III among all patients was 75.0%, 14.1%, and 10.9%, respectively. The frequency of preoperative and adjuvant chemotherapy were 0.8% (n = 25) and 19.2% (n = 572), respectively.

**Table 1:** Association of the patients’ clinicopathological characteristics with the postoperative 1 year ferritin-hemoglobin groups.

Factors	Total N=2,976 (%)	Postoperative 1 year ferritin-hemoglobin groups				*P* value
		Non-iron-deficiency without anemia (n=1,719)	Iron-deficiency without anemia (n=171)	Iron-deficient anemia (n=377)	Anemia of chronic disease (n=709)	
Age						< 0.001
< 60 years	1414 (47.5)	852 (49.6)	113 (66.1)	243 (64.5)	206 (29.1)	
≥ 60 years	1562 (52.5)	867 (50.4)	58 (33.9)	135 (35.5)	503 (70.9)	
Gender						< 0.001
Male	1912 (64.2)	1219 (70.9)	79 (46.2)	145 (38.5)	469 (66.1)	
Female	1064 (35.8)	500 (29.1)	92 (53.8)	233 (61.5)	240 (33.9)
ECOG						< 0.001
0 or 1	2870 (96.4)	1672 (97.3)	170 (99.4)	366 (97.1)	662 (93.4)	
2, 3, or 4	106 (3.6)	47 (2.7)	1 (0.6)	11 (2.9)	47 (6.6)
Smoking						< 0.001
None	1775 (59.6)	943 (54.9)	116 (67.8)	276 (73.2)	440 (62.1)	
Ex-smoker or current smoker	1201 (40.4)	776 (45.1)	55 (32.2)	101 (26.8)	269 (37.9)	
Approach method						< 0.001
Open	1207 (40.6)	600 (34.9)	83 (48.5)	186 (49.3)	338 (47.7)
Laparoscopy + Robot	1769 (59.4)	1119 (65.1)	88 (51.5)	191 (50.7)	371 (52.3)	
Extent of resection						< 0.001
Total gastrectomy	618 (20.8)	299 (17.4)	39 (22.8)	104 (27.6)	176 (24.8)	
Partial gastrectomy	2358 (79.2)	1420 (82.6)	132 (77.2)	273 (72.4)	533 (75.2)	
Anastomosis						0.266
Duodenal passing	502 (16.9)	307 (17.9)	30 (17.5)	53 (14.1)	112 (15.8)	
Duodenal non-passing	2474 (83.1)	1412 (82.1)	141 (82.5)	324 (85.9)	597 (84.2)	
Differentiation						< 0.001
Differentiated type	1396 (46.9)	831 (48.4)	80 (46.8)	129 (34.2)	356 (50.2)	
Undifferentiated type	1469 (49.4)	829 (48.3)	86 (50.3)	235 (62.3)	319 (45.0)	
Ect.	109 (3.7)	57 (3.3)	5 (2.9)	13 (3.4)	34 (4.8)	
Early postoperative complication						0.001
Absent	2332 (78.4)	1366 (79.5)	143 (83.6)	356 (80.9)	518 (73.1)	
Present	644 (21.6)	353 (20.5)	28 (16.4)	72 (19.1)	191 (26.9)	
TNM pStage*						< 0.001
I	2232 (75.0)	1430 (83.2)	124 (72.5)	241 (63.9)	437 (61.6)	
II	420 (14.1)	169 (9.8)	25 (14.6)	81 (21.5)	145 (20.5)	
III	324 (10.9)	120 (7.0)	22 (12.9)	55 (14.6)	127 (17.9)	

*According to the 8th edition of AJCC TNM classification.

The overall incidence of anemia was 36.5% (n = 1,086). The prevalence of ID and ACD was 18.4% (n = 548) and 23.8% (n = 709), respectively, at postoperative 1 year after gastrectomy. Among patients in the ID group, 68.8% (n = 377) had anemia. Overall, the prevalence of IDA was 12.7% (n = 377) at postoperative 1 year.

### 3.2 Relationship between clinicopathological characteristics and the patient groups

The relationships between the clinicopathological characteristics and anemia groups are summarized in **Table 1**. The prevalence rate of older age and higher ECOG grade were significantly higher in the ACD group (p < 0.001 and p < 0.001, respectively). Higher TNM stage was significantly associated with the ACD group (p < 0.001). The early postoperative complication rate was significantly higher in the ACD group than in the other groups (p = 0.001). IDA occurred significantly more frequently in female, non-smokers, patients with total gastrectomy and undifferentiated type (p < 0.001, p < 0.001, p < 0.001, and p < 0.001, respectively). The prevalence of partial gastrectomy was significantly higher in patients with ID without anemia (p < 0.001). The patients who underwent duodenal non-passing anastomosis tended to have IDA, but it was not significant (p = 0.266).

### 3.3 Prognostic factors of the anemia groups for OS, DFS and CSS

**Figure 1:**
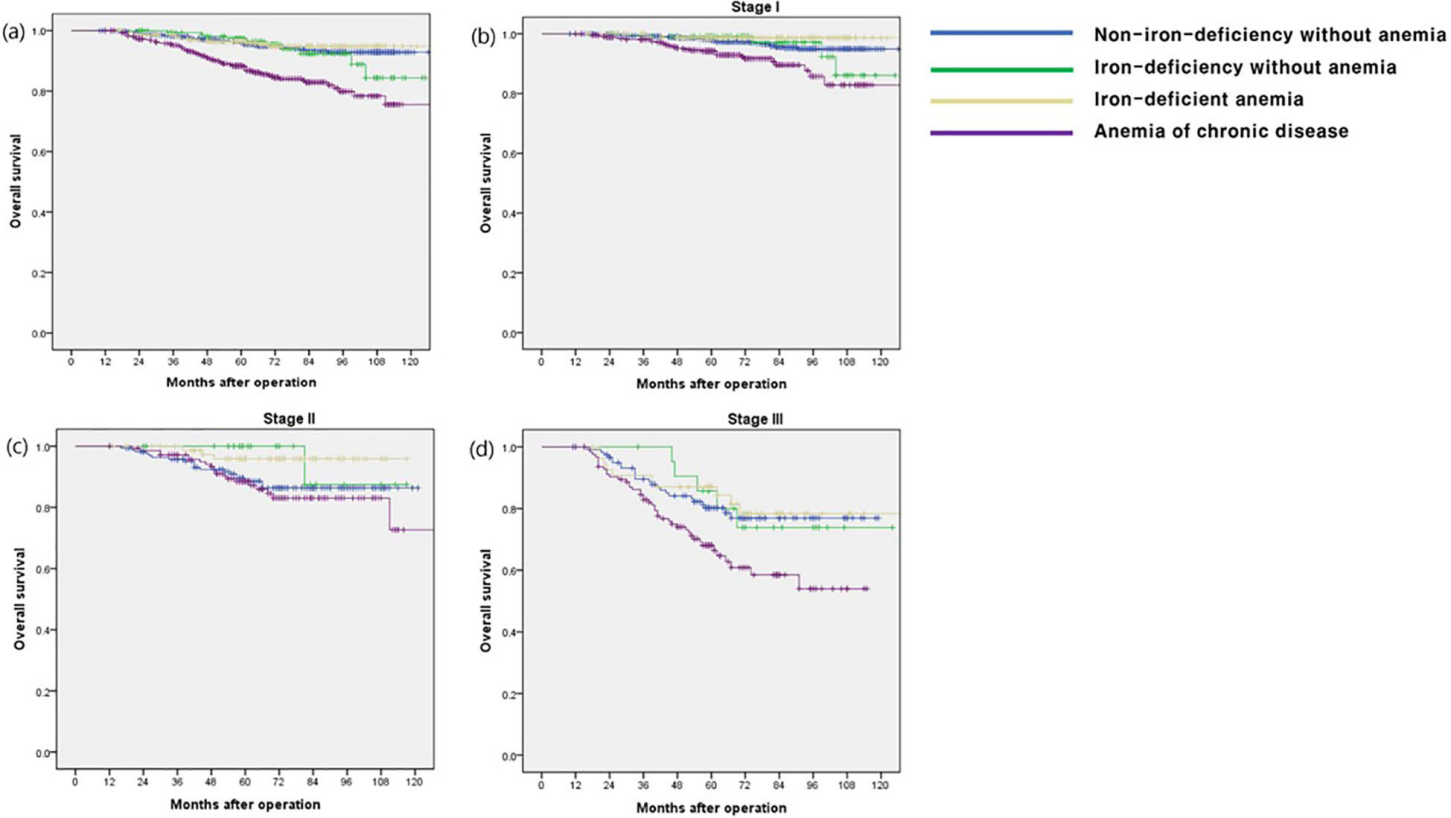
Overall survival according to postoperative 1 year ferritin-hemoglobin groups. **(a)** Total patients (p < 0.001), Non-iron-deficiency without anemia vs. Iron-deficiency without anemia (p = 0.704), Non-iron-deficiency without anemia vs. Iron-deficient anemia (p = 0.615), Non-iron-deficiency without anemia vs. Anemia of chronic disease (p < 0.001); **(b)** stage I (p < 0.001), Non-iron-deficiency without anemia vs. Iron-deficiency without anemia (p = 0.894), Non-iron-deficiency without anemia vs. Iron-deficient anemia (p = 0.170), Non-iron-deficiency without anemia vs. Anemia of chronic disease (p < 0.001); **(c)** stage II (p = 0.074) Non-iron-deficiency without anemia vs. Iron-deficiency without anemia (p = 0.268), Non-iron-deficiency without anemia vs. Iron-deficient anemia (p = 0.044), Non-iron-deficiency without anemia vs. Anemia of chronic disease (p = 0.560); **(d)** stage III (p = 0.009) Non-iron-deficiency without anemia vs. Iron-deficiency without anemia (p = 0.914), Non-iron-deficiency without anemia vs. Iron-deficient anemia (p = 0.663), Non-iron-deficiency without anemia vs. Anemia of chronic disease (p = 0.008).

**Figure 2:**
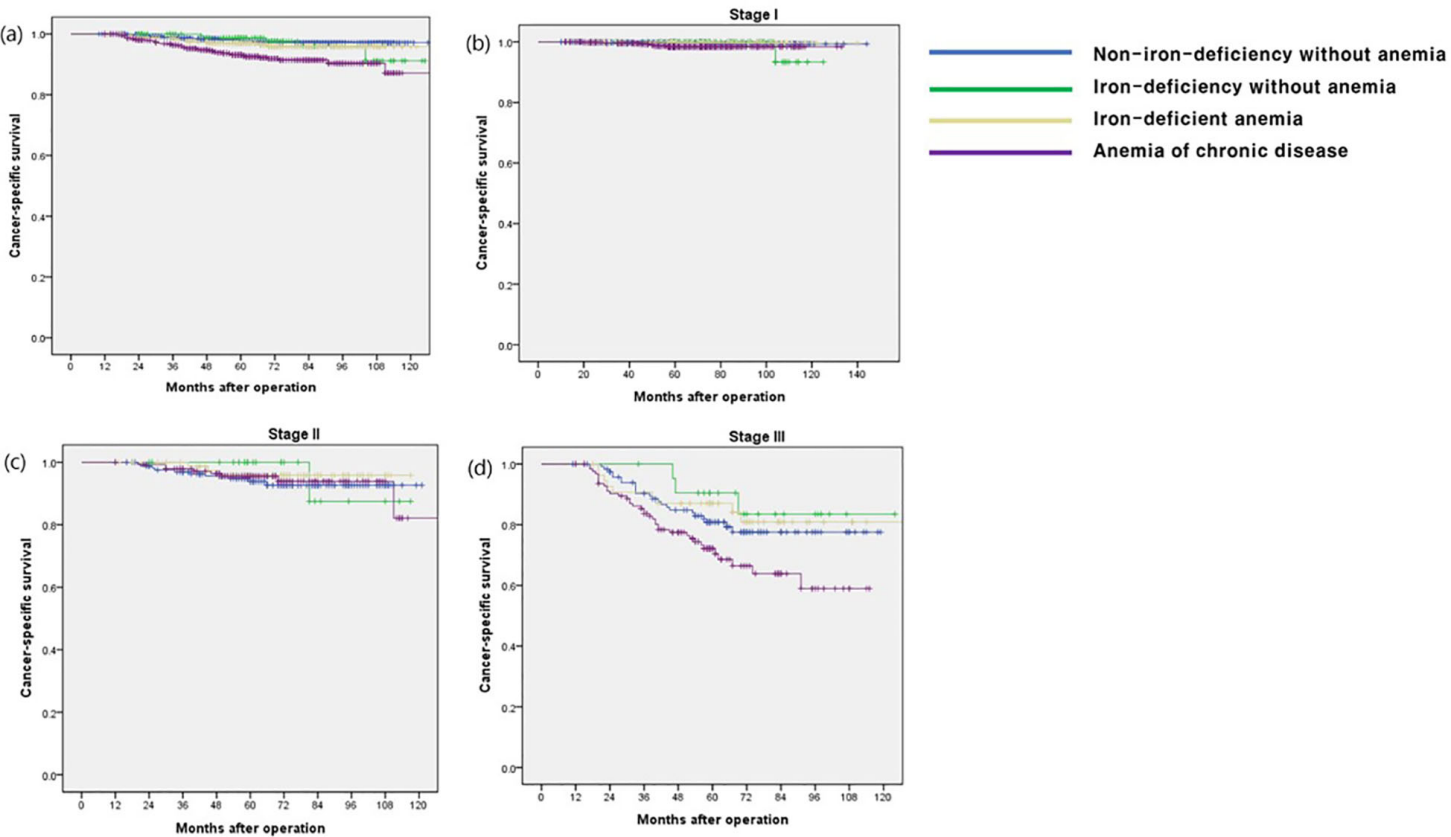
Cancer-specific survival according to postoperative 1 year ferritin-hemoglobin groups. **(a)** Total patients (p < 0.001) Non-iron-deficiency without anemia vs. Iron-deficiency without anemia (p = 0.697), Non-iron-deficiency without anemia vs. Iron-deficient anemia (p = 0.211), Non-iron-deficiency without anemia vs. Anemia of chronic disease (p < 0.001); **(b)** stage I (p = 0.124) Non-iron-deficiency without anemia vs. Iron-deficiency without anemia (p = 0.636), Non-iron-deficiency without anemia vs. Iron-deficient anemia (p = 0.979), Non-iron-deficiency without anemia vs. Anemia of chronic disease (p = 0.023); **(c)** stage II (p = 0.859), Non-iron-deficiency without anemia vs. Iron-deficiency without anemia (p =0.682), Non-iron-deficiency without anemia vs. Iron-deficient anemia (p = 0.411), Non-iron-deficiency without anemia vs. Anemia of chronic disease (p = 0.795); **(d)** stage III (p = 0.027) Non-iron-deficiency without anemia vs. Iron-deficiency without anemia (p = 0.392), Non-iron-deficiency without anemia vs. Iron-deficient anemia (p = 0.568), Non-iron-deficiency without anemia vs. Anemia of chronic disease (p = 0.035).

**Figure 3:**
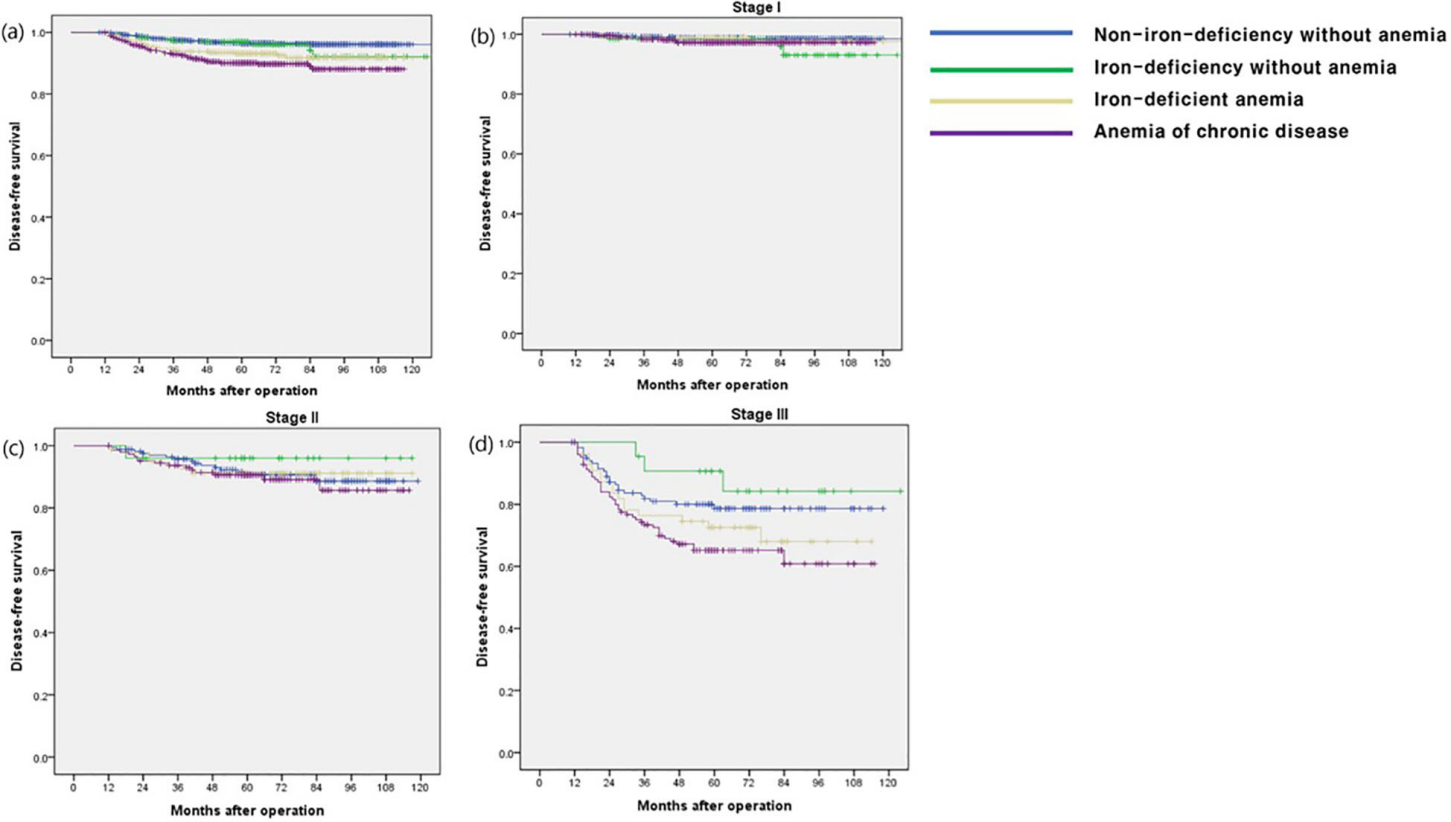
Disease-free survival according to postoperative 1 year ferritin-hemoglobin groups. **(a)** Total patients (p < 0.001) Non-iron-deficiency without anemia vs. Iron-deficiency without anemia (p =0.446), Non-iron-deficiency without anemia vs. Iron-deficient anemia (p = 0.001), Non-iron-deficiency without anemia vs. Anemia of chronic disease (p < 0.001); **(b)** stage I (p = 0.189) Non-iron-deficiency without anemia vs. Iron-deficiency without anemia (p = 0.118), Non-iron-deficiency without anemia vs. Iron-deficient anemia (p = 0.698), Non-iron-deficiency without anemia vs. Anemia of chronic disease (p = 0.070); **(c)** stage II (p = 0.772), Non-iron-deficiency without anemia vs. Iron-deficiency without anemia (p = 0.423), Non-iron-deficiency without anemia vs. Iron-deficient anemia (p = 0.908), Non-iron-deficiency without anemia vs. Anemia of chronic disease (p = 0.620); **(d)** stage III (p = 0.034) Non-iron-deficiency without anemia vs. Iron-deficiency without anemia (p = 0.379), Non-iron-deficiency without anemia vs. Iron-deficient anemia (p = 0.273), Non-iron-deficiency without anemia vs. Anemia of chronic disease (p =0.016).

The OS rate in the ACD group was significantly lower than that in the other groups (p < 0.001), especially in stage I and III disease (p < 0.001 and p = 0.009, respectively) (**Figure 1**). The CSS and DFS rates in the ACD group were significantly lower than those in the other groups (p < 0.001 and p < 0.001, respectively), especially for stage III disease (p = 0.034 and p = 0.027, respectively) (**Figures 2**, **3**).

In the multivariate Cox regression analysis, older age, higher ECOG grade, higher TNM stage, and ACD were independent risk factors for OS. In terms of CSS and DFS, higher TNM stage was an independent risk factor. In addition, the presence of ACD was a significant independent risk factor for OS [hazard ratio (HR) = 1.832; p < 0.001], CSS (HR, 1.690; p = 0.015), and DFS (HR, 1.714; p = 0.003) (**Tables 2**, **Supplementary Tables S1**, **S2**).

**Table 2:** Univariate and multivariate analyses of factors for the prediction of overall survival.

	Univariate analysis		Multivariate analysis	
	HR (95% CI)	*P* value	Adjusted HR (95% CI)	*P* value
Age				
< 60 years	Reference		Reference	
≥ 60 years	2.474 (1.827-3.351)	< 0.001	1.944(1.418-2.665)	<0.001
Gender				
Male	Reference		Reference	
Female	0.684 (0.504-0.930)	0.015	0.743 (0.544-1.015)	0.062
Smoking				
None	Reference			
Ex-smoker or current smoker	1.293 (0.981-1.705)	0.068		
ECOG				
0 or 1	Reference		Reference	
2, 3, or 4	3.023 (1.814-5.039)	< 0.001	2.174 (1.287-3.671)	0.004
Extent of resection				
Total gastrectomy	Reference			
Partial gastrectomy	0.581 (0.432-0.782)	< 0.001		
TNM stage*				
I	Reference		Reference	
II	2.944 (2.029-4.272)	< 0.001	2.588 (1.769-3.786)	<0.001
III	8.224 (6.031-11.214)	< 0.001	7.577 (5.502-10.436)	<0.001
Postoperative 1 year ferritin-hemoglobin groups				
Non-iron-deficiency without anemia	Reference		Reference	
Iron-deficiency without anemia	1.142 (0.592-2.204)	0.691	0.955 (0.492-1.855)	0.893
Iron-deficient anemia	0.868 (0.507-1.483)	0.604	0.689 (0.398-1.193)	0.184
Anemia of chronic disease	3.100 (2.306-4.167)	< 0.001	1.832 (1.342-2.501)	<0.001

*According to the 8th edition of AJCC TNM classification; HR, hazard ratio; CI, confidence interval.

### 3.4 Risk factors for the occurrence of ACD

In multivariate logistic regression analysis, older age and higher TNM stage were independent risk factors for ACD (p < 0.001 and p < 0.001, respectively). The patients with lower PNI, presence of preoperative anemia, and early postoperative complications were significant risk factors for ACD (p = 0.026, p < 0.001, and p = 0.008, respectively) (**Table 3**).

**Table 3:** Univariate and multivariate analyses of factors for the prediction of anemia of chronic disease.

	Univariate analysis		Multivariate analysis	
	OR(95% CI)	*P* value	Adjusted OR(95% CI)	*P* value
Age				
< 60	Reference			
≥ 60	2.785 (2.322-3.341)	<0.001	2.468 (1.993 -3.056)	<0.001
Gender				
Male	Reference			
Female	0.896 (0.750-1.070)	0.226		
ECOG				
0 or 1	Reference			
2, 3, or 4	2.657 (1.794-3.936)	<0.001		
Properative BMI				
< 18.5	Reference			
≥ 18.5, < 25.0	0.902 (0.590-1.379)	0.691		
≥ 25.0	0.759 (0.489-1.176)	0.257		
Preoperative PNI group				
Low	Reference			
High	0.484 (0.398-0.589)	<0.001	0.779 (0.626-0.970)	0.026
Preoperative anemia				
Absent	Reference			
Present	3.711 (3.069-4.487)	<0.001	2.487 (1.954-3.167)	<0.001
TNM stage*				
I	Reference			
II	2.166 (1.726-2.717)	<0.001	1.888 (1.433-2.472)	<0.001
III	2.648 (2.070-3.388)	<0.001	2.058 (1.536-2.758)	<0.001
Extent of resection				
Total	Reference			
Partial	0.733 (0.601-0.895)	0.002		
Anastomosis				
Duodenal passing	Reference			
Duodenal non-passing	1.108 (0.880-1.393)	0.383		
Early postoperative complication				
Absent	Reference			
Present	1.477 (1.215-1.795)	<0.001	1.376 (1.089-1.738)	0.008

*According to the 8th edition of AJCC TNM classification; OR, odds ratio; CI, confidence interval; BMI, body mass index; PNI, prognostic nutritional index.

### 3.5 Correlation between ACD and preoperative nutritional and inflammatory condition

The correlations between ACD and preoperative nutritional and inflammatory conditions are summarized in **Table 4**. In patients with ACD at postoperative 1 year, the total protein, albumin, triglyceride, low-density lipoprotein cholesterol, high-density lipoprotein cholesterol, Hb, iron, and calcium levels were significantly lower than the preoperative laboratory results (p < 0.001, p < 0.001, p = 0.001, p < 0.001, p = 0.006, p < 0.001, p = 0.026, and p < 0.001, respectively). Systemic inflammatory markers such as NLR and PLR were not significantly related to ACD occurrence (**Table 4**).

**Table 4:** Correlation between anemia of chronic disease and nutritional and inflammatory condition.

Prepoperative factors	Anemia of chronic disease at postoperative 1 year		*P* value
	Absent	Present	
Total protein	7.02 ± 0.53	6.85 ± 0.62	<0.001
Albumin	4.3 ± 0.37	4.08 ± 0.45	<0.001
Triglyceride	123.76 ± 88.00	111.15 ± 60.66	0.001
Total cholesterol	181.24 ± 36.12	175.18 ± 40.28	0.104
LDL cholesterol	112.65 ± 31.48	105.91 ± 31.59	<0.001
HDL cholesterol	48.38 ± 12.87	46.73 ± 11.82	0.006
Hemoglobin	14.05 ± 3.04	12.80 ± 1.49	<0.001
Iron	81.72 ± 47.78	72.51 ± 44.94	0.026
Ferritin	87.04 ± 84.30	85.87 ± 96.95	0.902
TIBC	311.80 ± 56.89	299.58 ± 58.70	0.073
Folate	10.31 ± 4.52	10.61 ± 5.21	0.578
Vitamin B12	587.61 ± 298.95	563.43 ± 315.60	0.486
Calcium	9.09 ± 0.43	8.97 ± 0.49	<0.001
Phosphorous	3.53 ± 0.49	3.54 ± 0.57	0.623
NLR	2.02 ± 1.21	2.08 ± 1.11	0.278
PLR	134.56 ± 58.34	134.56 ± 49.91	0.997

NLR, neutrophil-to-lymphocyte ratio; PLR, platelet-to-lymphocyte ratio.

## 4 Discussion

This study highlights a notable incidence of post-gastrectomy anemia, with a higher prevalence of ACD than IDA. Patients diagnosed with ACD tended to be older, had higher ECOG scores, experienced more early postoperative complications, and frequently had a more advanced stages of gastric cancer than the other groups. Importantly, the presence of ACD emerged as a significant risk factor for poor long-term OS, particularly evident in stages I and III.

Various manifestations of post-gastrectomy anemia in gastric cancer include association with morbidity and mortality, decreased quality of life, and poor functional status among patients with cancer (5, 17). Anemia impairs quality of life by causing a variety of symptoms ranging from mild headache and fatigue to impaired work capacity to cognitive impairment (5, 18).

In this study, the overall incidence of anemia was 36.6%, with IDA and ACD prevalences of 12.7% and 23.9%, respectively, at postoperative 1 year. Approximately eight studies had defined post-gastrectomy anemia and reported its incidence (5, 7, 8, 19–23). Similar to the current study, only two out of approximately eight studies specifically subclassified post-gastrectomy anemia and reported its detailed incidence (5, 20). According to the study published in 2012, the risk of anemia increases with time and affected 27% of patients at 1 year and 37% at 2 years after gastrectomy among patients with early gastric cancer (20). In this study, IDA predominated at approximately 12%, with ACD being < 5%. The discrepancy in the occurrence rates of ACD in our study is likely attributable to differences in the established ferritin cutoff values. Their study differed from ours in that it focused exclusively on anemia in early gastric cancer. In our study, at postoperative 1 year, 30.5% of stage I patients experienced anemia, showing similar results upon detailed analysis (data not-shown). In a study conducted in 2016, the occurrence of anemia was reported from 1 to 5 years post-gastrectomy, distinguishing only between IDA and vitamin B12 deficient anemia. The incidence of anemia was reported to be 18.7% at postoperative 1 year, with < 10% of cases with IDA, excluding preoperative anemia patients from the analysis (5).

Furthermore, other studies have reported heterogeneous incidence rates of anemia (7, 22). For instance, one study in 2019 reported a 27.4% incidence of anemia at 1 year post-gastrectomy (22), whereas another study in 2018 focusing solely on patients with stage I gastric cancer for up to 5 years post-surgery reported a 7.1% incidence of anemia at 1 year post-surgery (7). Additionally, other studies have reported post-gastrectomy incidence rates according to sex, extent of gastric resection, and reconstruction method (8, 19, 21). Exclusion criteria varied across studies, contributing to the observed heterogeneous results.

Interestingly, most of these studies have shown considerable interest in the incidence of anemia based on the reconstruction methods (7, 21, 24–26). Among them, Imamura et al. found that the reconstruction method independently influences post-gastrectomy anemia, with type Billroth-I anastomosis associated with less decline in Hb levels (24). While our study also examined anastomosis types, we did not observe any significant differences.

Several studies have analyzed anemia as a prognostic factor in gastric cancer (7, 27–31). However, most studies tend to focus on preoperative or pre-treatment anemia, with limited postoperative data available (27–31). To the best of our knowledge, only one previous study has investigated the correlation between post-gastrectomy anemia and long-term survival in patients with gastric cancer. In that study, patients with anemia had a significantly poorer long-term prognosis. However, the study did not provide detailed data for each anemia type (7).

In our study, ferritin level was an important parameter for the classification of anemia subtypes. Ferritin is the principal iron-binding protein, a transporter, and a recycler of iron (9, 10). Ferritin serves as a reservoir for iron, thus a decrease in its levels reflects iron deficiency, while elevated ferritin levels typically signify the presence of ACD (9, 10, 32). Ferritin not only aids in iron transport and recycling but also plays a role in cellular defense against oxidative stress and inflammation (10, 33). Increased serum ferritin levels are associated with various conditions including liver diseases, infections, inflammatory conditions, and malignancy (9, 10, 33). In particular, ferritin can be produced by the tumor microenvironment in solid tumors (10). Therefore, ferritin is considered an oncofetal protein and has been implicated as a tumor marker in lung, breast, and renal cancers (34–36). In recent years, there has been an increased interest in ferritin as a marker of iron overload, reflecting oxidative stress, the pro-inflammatory role of iron in carcinogenesis, and pathological nutritional status (9, 10, 32, 33, 37). Furthermore, serum ferritin levels have been suggested to have diagnostic value and are also useful for evaluating treatment efficacy and prognosis in various cancer (11–14, 34–36, 38). However, the prognostic role of serum ferritin in gastric cancer remains controversial. Approximately only two studies have demonstrated the prognostic role of ferritin in gastric cancer (15, 16). One study compared pre- and post-treatment ferritin levels in patients with gastric cancer, observing an increase in ferritin levels in non-responsive cases and a significant decrease in responsive cases (16). Another study found a positive correlation between serum ferritin levels and sarcopenia, and a high serum ferritin level was an independent poor prognostic factor (15). Recent studies have suggested that combining multiple tumor markers may improve the prognostic accuracy for gastric cancer patients. For instance, a study demonstrated that a combination of three important tumor markers—CEA, CA72-4, and CA19-9—provided significantly better prognostic value than individual markers alone (39). In addition, emerging research also emphasizes the role of BMI in the prognosis and treatment response of cancer patients (40). This suggests that a multifactorial approach, incorporating ferritin along with other biomarkers, may provide more robust insights into the long-term prognosis of gastric cancer patients in the future.

Several studies have contributed to our understanding of the association between elevated ferritin levels and poor prognosis in gastric cancer (15, 41). A 2020 study concluded that iron overload may be related to muscle loss in patients with gastric cancer cachexia and that elevated ferritin levels are associated with muscle wasting (41). In a 2023 study, preoperative serum ferritin levels were associated with sarcopenia and the prognosis of gastric cancer (15).

Other studies on gastric cancer have explored the different aspects of ferritin levels (42–44). In 2023, a systematic review and meta-analysis investigated the diagnostic significance of ferritin levels in gastric cancer (42). A 2020 study examined postoperative ferritin levels as a nutritional factor (43), whereas a 2022 study identified postoperative ferritin deficiency as a common complication in patients with gastric cancer, analyzing the predictive factors for ferritin deficiency (44).

Our study has several strengths. First, it was based on reliable large-scale data collected from multiple centers over an extended period. Second, parameters such as Hb and ferritin, which were presented in our study, were routinely assessed during follow-up, requiring no additional effort to evaluate. These markers are easy to measure, minimally invasive, and cost effective. Third, our study makes a significant contribution by classifying post-gastrectomy anemia types and elucidating their association with long-term prognosis in gastric cancer patients. We found that the classification of anemia is crucial for identifying factors that influence long-term outcomes. Specifically, our detailed categorization based on hemoglobin and ferritin levels provides valuable insights into the prognostic implications of anemia, highlighting its potential utility as a marker for monitoring gastric cancer progression after gastrectomy. This nuanced approach to anemia classification represents a key innovation of our study, offering a novel perspective that may improve clinical management and patient outcomes. Fourth, our study provides valuable clinical insights into early interventions for high-risk patients, particularly emphasizing the importance of nutritional support, such as iron supplementation or dietary adjustments, to prevent further anemia progression and improve long-term outcomes. Additionally, we highlight the need for close monitoring, including radiologic and tumor marker assessments, to detect potential recurrence in high-risk patients. These findings contribute to a more comprehensive approach to managing post-gastrectomy anemia and improving the overall prognosis of gastric cancer patients. Therefore, Hb and ferritin levels may serve as valuable alternative markers for monitoring disease progression during follow-up. Lastly, our study uniquely concentrated on ACD in post-gastrectomy patients and investigated its impact on long-term survival, analyzing the risk factors for ACD occurrence for the first time to our knowledge. This enables clinicians to identify and prevent efforts aimed at understanding and preventing the risk factors for the development of ACD during long-term postoperative follow-up.

Despite its strengths, our study has several limitations. First, owing to its retrospective nature, inherent biases may have influenced the findings. Second, the lack of standardized protocols and the variability in anemia management, combined with the retrospective design of this study, limited the ability to control for treatment variables related to postoperative anemia, such as iron supplementation and blood transfusion, which may have influenced the postoperative 1-year anemia status of patients. Due to the retrospective study, we encountered challenges in systematically tracking the exact timing and details of iron supplementation or blood transfusions after surgery. While some patients may have received treatment for anemia, based on clinical discretion, we were unable to collect comprehensive data on these interventions for all patients. Third, the absence of time-dependent serial tests for post-gastrectomy anemia represents another limitation. Finally, this study relied on limited laboratory results for anemia. In particular, vitamin B12 results was excluded from the results of this study. There have been investigations into the role of total iron-binding capacity and hematocrit levels in gastric cancer prognosis. Therefore, comprehensive, well-designed prospective studies are warranted to address these limitations and enhance our understanding of post-gastrectomy anemia in patients with gastric cancer in the future.

## 5 Conclusion

The relationship between ferritin and Hb at postoperative 1 year are significant prognostic factor for overall survival in patients with gastric cancer. In particular, the presence of ACD can be a more specific predictor of gastric cancer. Therefore, clinicians should pay attention to and correct ACD in outpatient clinics during postoperative follow-up.

## Data Availability

The raw data supporting the conclusions of this article will be made available by the authors, without undue reservation.
