# Supplementary material for: Post-gastrectomy anemia and ferritin dynamics: key determinants of prognosis and clinical management in patients with gastric cancer
Source: Front Oncol. 2025 Mar 14;15:1487477. doi: 10.3389/fonc.2025.1487477 (PMC11949920; doi:10.3389/fonc.2025.1487477)
Supplement: Supplementary file 1 [file DataSheet1.doc]

Table S1. Multivariate analyses of factors for the prediction of cancer-specific survival

|  | Multivariate analysis | | |
| --- | --- | --- | --- |
|  | Adjusted HR(95% CI) | *P* value |  |
| TNM stage* |  |  | |
| I | Reference |  | |
| II | 7.833 (3.976-15.430) | <0.001 | |
| III | 36.905 (20.609-66.087) | <0.001 | |
| Postoperative 1 year ferritin-hemoglobin groups |  |  | |
| Non-iron-deficiency without anemia | Reference |  | |
| Iron-deficiency without anemia | 0.705 (0.277-1.795) | 0.464 | |
| Iron-deficient anemia | 0.771 (0.410-1.450) | 0.420 | |
| Anemia of chronic disease | 1.690 (1.106-2.583) | 0.015 | |

* According to the 8th edition of AJCC TNM classification; HR, hazard ratio; CI, confidence interval

Table S2. Multivariate analyses of factors for the prediction of disease-freel survival

|  | Multivariate analysis | | |
| --- | --- | --- | --- |
|  | Adjusted HR(95% CI) | *P* value |  |
| TNM stage* |  |  | |
| I | Reference |  | |
| II | 4.905 (3.106-7.747) | <0.001 | |
| III | 16.232 (10.968-24.021) | <0.001 | |
| Postoperative 1 year ferritin-hemoglobin groups |  |  | |
| Non-iron-deficiency without anemia | Reference |  | |
| Iron-deficiency without anemia | 0.871 (0.415-1.829) | 0.715 | |
| Iron-deficient anemia | 1.271 (0.800-2.018) | 0.310 | |
| Anemia of chronic disease | 1.714 (1.198-2.453) | 0.003 | |

* According to the 8th edition of AJCC TNM classification; HR, hazard ratio; CI, confidence interval
